# Refining the DC-targeting vaccination for preventing emerging infectious diseases

**DOI:** 10.3389/fimmu.2022.949779

**Published:** 2022-08-09

**Authors:** Yadira Pastor, Nour Ghazzaui, Adele Hammoudi, Mireille Centlivre, Sylvain Cardinaud, Yves Levy

**Affiliations:** ^1^ Vaccine Research Institute, Université Paris-Est Créteil, Institut Mondor de Recherche Biomédicale, Inserm U955, Team 16, Créteil, France; ^2^ Assistance Publique-Hôpitaux de Paris, Groupe Henri-Mondor Albert-Chenevier, Service Immunologie Clinique, Créteil, France

**Keywords:** dendritic cells, vaccine, viral infection, epitope mapping, SARS–CoV–2

## Abstract

The development of safe, long-term, effective vaccines is still a challenge for many infectious diseases. Thus, the search of new vaccine strategies and production platforms that allow rapidly and effectively responding against emerging or reemerging pathogens has become a priority in the last years. Targeting the antigens directly to dendritic cells (DCs) has emerged as a new approach to enhance the immune response after vaccination. This strategy is based on the fusion of the antigens of choice to monoclonal antibodies directed against specific DC surface receptors such as CD40. Since time is essential, *in silico* approaches are of high interest to select the most immunogenic and conserved epitopes to improve the T- and B-cells responses. The purpose of this review is to present the advances in DC vaccination, with special focus on DC targeting vaccines and epitope mapping strategies and provide a new framework for improving vaccine responses against infectious diseases.

## Introduction

Vaccination is the most successful and cost-effective contribution for infectious disease prevention and the control of major pathogens which threat public health. It is estimated that around 3 million lives are saved every year by the current immunization plans, with 28 vaccines available for human use (1). However, there are still both well-stablished and emerging diseases for which the development of successful vaccines is still a challenge.

Although the incidence of infectious diseases has decreased in the last decades, they are still contributing to major health and economic costs. For several widespread and life-threatening infectious diseases such as HIV, tuberculosis (TB), HBV or influenza, an effective long-term protective vaccine is still lacking. These diseases, together with emerging and reemerging pathogens, increase the list of high priority diseases that urgently need prophylactic or therapeutic immunotherapies.

More than ten major viral disease epidemics or pandemics have affected human population in the last century, posing a considerable risk for an international public health emergency, due to their potential to spread rapidly (2). Emerging diseases constitute at least 15% of all human pathogens and are caused mostly by zoonotic pathogens. Among them, avian/bird flu, Swine flu, Middle East respiratory syndrome coronavirus (MERS-CoV), Severe acute respiratory syndrome (SARS), Crimean Congo haemorrhagic fever (CCHF), Lassa fever, Rift Valley fever (RVF), Marburg virus disease, Ebola, Zika, Nipah and Henipaviral diseases have originated sporadic or repeated outbreaks which needed a rapid intervention by the governments and scientific community (3).

Unlike other human diseases, infectious diseases might have unpredictable behavior, with potential to cause global outbreaks and pandemics. Although many of these diseases might be preventable with the use of prophylactic or therapeutic immunotherapies which can offer a rapid response against the pathogens, there is an unmet vaccine need for many of these infectious threats. Therefore, the development of new and alternative strategies to respond to the potential emerging diseases effectively and rapidly is necessary.

In the race of obtaining good vaccine candidates against these pathogens, a wide range of different platforms have been developed in the recent years which offer more robust immune responses and scalable manufacturing comparing to conventional vaccines based on live attenuated or inactivated vaccines, which may be adapted and applied across multiple pathogens. These strategies include nucleic acid, viral-vector or recombinant protein-based vaccines (3).

These new approaches try to deal with immunological challenges, such as the high genetic variability of many pathogens such as HIV, HCV or influenza viruses, or the limited understanding of the required immune response for some diseases (4–6). Thus, these new strategies can improve the antigen delivery and its presentation to adaptive immune cells, including both B- and T-cell responses required for an effective protection. Additional tools for the vaccine development include i) the bioinformatics immunogen design and protein engineering, ii) the cell sorting and sequencing technologies that allow single-cell analysis of the immune responses, and iii) the genetically modified animal models for the vaccine testing (7).

Some of these new vaccine platforms have been very well-stablished during the last years, such as the gene-based vaccine platforms, especially nucleic acid and viral vector-based vaccines, which have already shown their safety and efficacy against influenza (8), Zika (9), Ebola (10), Chikungunya (11) or more recently, against SARS-CoV-2 virus (12). Others, like recombinant protein design, represent a safe and low-cost design platform, which allows an efficient antigen delivery and face the challenge of genetic diversity by choosing the most immunogenic conserved regions of the pathogen (13). The antigenic epitopes can be displayed in high copy number, and they may have the same characteristics as the original pathogen, being able to induce a high T- and B-cell immune response. Vaccines based on recombinant proteins have shown to be effective against several viral infections, such as HBV (14) or human papillomaviruses (HPVs) (15), among others, inducing high titers of virus-neutralizing antibodies and T-cell responses.

However, for the previously mentioned or other intracellular pathogens, obtaining a strong and long-lasting immune response is sometimes a challenge and thus, alternative or a combination of the previous strategies to improve the adaptive response are being developed.

Among them, the targeting of immunogens to antigen-presenting cells (APC) such as dendritic cells (DCs), has demonstrated to be a powerful tool to induce the clonal expansion of specific B- and T-cells (16). This is critical in those diseases that require cellular immunity, such as diseases caused by intracellular pathogens, major chronic infections including viral hepatitis, AIDS, human papillomavirus-linked pathologies, tuberculosis and more recently as a boost of first generation of COVID-19 vaccines as well as in other non-infectious diseases like cancer (17). Prophylactic DC-based approach, inducing CD4^+^ and CD8^+^ T-cells responses in addition to neutralizing antibodies appears as a promising scheme to get a full and long-term protection, while minimizing any risk of viral escape mechanism.

## DC-based vaccines against infectious diseases

### Immunological functions of DC

DC are professional APC that drive the immune system responses by sensing, processing and presenting the pathogens to naïve T-cells, inducing the activation and differentiation of effector lymphocytes (18). They are characterized by a high expression of major histocompatibility complex class II molecules (MHC-II) and CD11c -although many other markers are present- allowing the classification into different subtypes. Moreover, DC play a tolerogenic role depending on their microenvironment (19), which is a critical immune function to prevent any immune-mediated tissue damage.

When exposed to infectious antigens, immature DC recognize specific ligands through pattern-recognition receptors (PRR), such as toll-like receptors (TLR) and C-type Lectin receptors (CLR), which are differently expressed on the different DC-subsets, and migrate to secondary lymphoid organs where the T-cell presentation occurs (20). During this process, DC upregulate chemokine receptors like CCR7 and produce cytokines supporting T-cell activation and differentiation. Specifically they induce differentiation of CD4^+^ T-cells into T-regulatory (Treg) or T-helper subsets (Tfh, Th1, Th2, Th9, Th17 and Th22) (21). DCs are also critical for B-cell proliferation and antibody synthesis by producing soluble factors, such as IL-12. They orchestrate thus the isotopic recombination, playing a direct role on the differentiation and fate of activated B-cells and the organization of primary B-cell follicles (22). Resident lymphoid tissue DCs remain in the draining lymph nodes during their entire life, while non-lymphoid organ tissue DCs migrate continuously from peripheral organs to the draining lymph nodes, either spontaneously under steady-state conditions or upon inflammation-induced activation in a CCR7-dependent manner (23). Importantly, DCs have cross-presentation ability, being able to present extracellular antigens through the MHC class I (MHC-I), necessary for the cytotoxic immune response driven by antigen specific CD8^+^ T-cells (24). This cross-presentation process is critical in the context of vaccination since it allows DCs to prime CD8^+^ in the absence of CD4^+^ T lymphocytes.

### DC subsets and immune response

**Figure 1:**
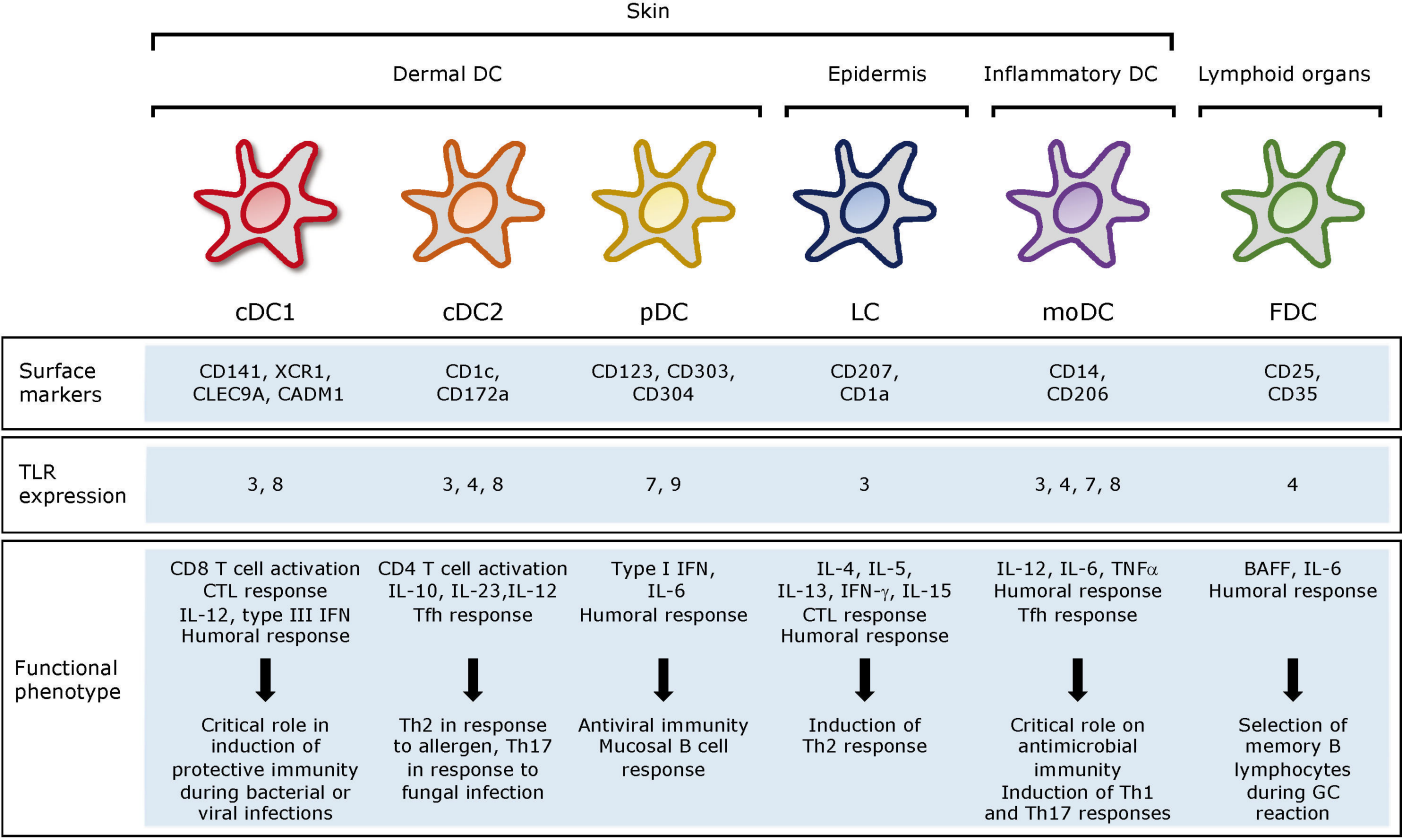
Subsets, location and function of human DC. cDC, conventional DC; pDCs, plasmacytoid DC; LC, Langerhans cells; moDC, monocyte derived DC; FDC, follicular DC; CTL, cytotoxic T lymphocytes; Tfh, T follicular helper.

The great complexity and plasticity in phenotype and functionality have made difficult to precisely classify the human DC and it is continuously under revision. *Ex vivo* isolated DC demonstrated that each DC subset promote specific immune function (25). In general, DCs can be divided into resident lymphoid tissue DCs and migratory non-lymphoid tissue DCs, which in turn, can be divided into other subsets depending on the surface markers and functions they have (**Figure 1**, adapted from Cohn L et al., 2014) (26).

In the bloodstream, we can find both conventional DCs (cDC), which can be subdivided into cDC1 and cDC2, and plasmacytoid DCs (pDC), which can migrate from the blood to lymphoid and non-lymphoid tissues. cDCs are defined by zBTB46 transcription factor expression (27). They are present in almost all tissues and the most abundant DC subset. Whether they are in lymphoid or non-lymphoid tissues and what is required for their development, cDCs can be classified into different subsets using a unified classification strategy (23). The cDC1 subpopulation can be identified by their surface expression of XCR1 or CD141, c-type lectin CLEC9A and CADM1 cell adhesion molecule. Myeloid cDC1 are characterized by their intrinsic capacity to cross present antigens *via* MHC-I to activate CD8^+^ T-cells and to promote Th1 and NK responses through IL-12 secretion. This secretion is particularly low compared to adequately activated cDC2. Although controversial, it is in line with the capacity of these cells to interact and present antigens to Th1 cells (28). cDC2 in turn, express CD1c and CD172α and cover many roles in the immune system regulation, being major inducers of Th2 and Th17 responses and therefore being essential against extracellular pathogens (29). The pDCs can be of lymphoid or myeloid origin and are identified by their expression of CD123, CD303 and CD304 while lacking CD11c. They are characterized by the rapid and high-level secretion of type I interferon (IFN), including IFN-α and –β, upon TLR7 and TLR9 stimulation and therefore are particularly important in viral infections (30).

Langerhans cells (LCs) are a type of DC residing in the epidermis of the human skin (31). As such, they are key regulators of immune function and have been considered as prime targets for novel transcutaneous vaccines. Essentially, the induction of protective T-cell immunity by these vaccines requires the efficient and specific delivery of pathogen-associated antigens to LC (32). Human LCs express Langerin (CD207) receptor, an endocytic C-type lectin receptor as well as CD1a (33). This, together with the low levels of CD11b and CD11c, allows discriminating with the dermal cDC2.

In the context of a skin infection, local production of TNFα and IL-1β activate LCs that migrate to secondary lymph nodes. When activated, they produce high levels of IL-15 and stimulate CD8^+^ T-cells (34). In addition, LCs and cDC1 can regulate the humoral immune responses through the differentiation of distinct T follicular helper (Tfh) cells (35). Thus, LCs induce germinal center (GC)-dependent antibody responses in the absence of an adjuvant (36). First, LCs stimulate the formation of Tfh cells, and then migrate to the B-cell region to initiate B-cell responses. Certain mechanisms can inhibit these GC responses induced by LCs including the delivery of IL-10, high antigen dose and co-delivery of antigen to cDC1 (37).

Unlike other subpopulations of DCs, monocyte-derived DCs (moDC) arise from monocytes recruited into tissues under inflammatory conditions by populating quickly the site of infection and initiate CD8^+^ T-cells responses by antigen cross presentation (38). They can be derived *in vitro* by stimulation of CD34^+^ precursor with GM-CSF and TNFα, as well as with GM-CSF and IL-4 if expanded from monocytes (39). This makes them a great tool to characterize the DC responses *in vitro*, as well as to use them as tools to generate therapeutic vaccines (21).

Note that follicular dendritic cells (FDCs) are non-hematopoietic cells of stromal origin, indispensable for efficient GC- formation in lymphoid organs. They are located within B-cell follicles and in the light zones of germinal centers, and are crucial for hypermutated specific B-cells maturation and fate, promoting them to switch and produce high-affinity antibodies and B-cell memory (40). FDCs secrete cytokines fostering GC B-cells survival such as IL-6 and BAFF (41).

In the context of an infection, it is of great importance to consider the mucosal tissues and the mucosal immune response. There are different immune cells present in mucosal barriers, including DC or macrophages, which maintain the homeostasis within the host facing exogenous antigens, by presenting them and inducing T-cell responses and IgA production (42). In addition to resident DCs, other APCs are recruited in the site of infection, contributing to protective responses. Two main DC populations have been identified in gut-draining lymph nodes: CD103^+^CD11b^-^ DCs and CD103^+^CD11b^+^ DCs, which lead to tolerogenic or pro-inflammatory responses, respectively (42). Mucosal vaccines might also contribute to enhance APC in the site of administration, thus enhancing adaptive immunity. Interestingly, different gene expression in DC populations has been reported depending on the different regions within the gut and mucosal surfaces, indicating that not only the subset but also the location of DC might play a critical role for the type of immune response generated after vaccination (43). Moreover, with the aim of recruiting DC, some mucosal vaccines use pro-inflammatory molecules or adjuvants, which trigger inflammation on the site of administration and promote T-cell priming mediated by moDC.

### DC-based approaches against infectious diseases

Different strategies have been studied for the vaccination with DC. Due to their good properties for inducing T-cell immune response, most of these strategies have been focused on cancer immunotherapies, although some have been also evaluated for different infectious diseases. These strategies can be grouped in two main approaches: the *ex vivo* peptide-loaded DC vaccines and the *in vivo* DC-targeting vaccines.

#### *Ex vivo* peptide-loaded DC

This strategy aims to generate and expand autologous DCs from the patient *ex vivo*, loaded with specific antigen from the tumor or pathogen, and then re-introduce them into the patient (44). In order to do this, DCs can be isolated either from the peripheral blood of the patient or from *in vitro* differentiation from monocytes or CD34^+^ hematopoietic cells in the presence of GM-CSF and IL-4 (45). DCs are then loaded with the antigen, either by direct incubation with them or by fusion to the specific tumor cells. Cells are finally maturated with different molecules such as LPS, IFN-γ or TNFα before introducing to the patient (44). Many clinical assays have been performed using this technique with very promising results (46, 47). Indeed, US FDA approved the first DC-immunotherapy for advanced metastatic prostate cancer (sipuleucel-T, Provenge^®^), based on autologous cells expanded *ex vivo* in the presence of a prostatic acid phosphatase/GM-CSF fusion protein (48).

Regarding infectious diseases, vaccine candidates based on this *ex vivo* approach have shown effectiveness in murine models and clinical assays against parasite diseases such as visceral leishmaniasis (49, 50), against fungi such as *Candida albicans* (51) or *Cryptococcus gattii* (52), or against viral infections such as Influenza (53), Herpes simplex virus (54, 55) or HIV (46, 56). In this setting, DCs loaded with HIV-derived long lipopeptides covering Gag, Nef and Pol epitopes (LIPO-5-DC vaccine) induced polyfunctional HIV-specific responses that were negatively correlated with the maximum viral load after HAART cessation (47). Recently, a Phase I-II clinical trial has been approved against SARS-CoV-2 infection, which includes 175 participants, consisting of autologous DCs previously loaded *ex vivo* with SARS-CoV-2 spike protein, with or without GM-CSF, to prevent COVID-19 infection in adults, although no data is available yet (57).

DC-based therapies based on *ex vivo* loaded-DCs have the main advantage to be a fully controlled vaccine system. The cells are centrifuged, submitted for culture, activated, and then returned to the patient as an immune modulator or vaccine. Nonetheless, this medical approach must be personalized involving heavy and costly procedures. Hence, the development of universal anti-DC mAbs, that can be used for any patient, appears as a constructive alternative.

#### *In vivo* targeting of DC

The delivery of antigens directly to DCs *in vivo* can be achieved by coupling antigens to monoclonal antibodies (mAb) that recognize specific DC surface molecules. This approach offers some advantages comparing to *ex vivo* strategies, since it does not manipulate the DC, which are usually sensitive to experimental handling and can show changes in the DC activation phenotype comparing to their natural *in vivo* phenotype (58). Furthermore, they are safe and scalable vaccine products that may decrease the vaccine dose and, since it drives to specific cells, avoids unspecific targeting reducing adverse effects (17).There are several ways to couple the antigens to the mAb. One option is to combine the antibodies with nanoparticles (or liposomes) containing either the antigen of choice or the DNA encoding such antigen, with ligands or antibodies that bind specifically to DC surface receptors (59). This strategy, specially the use of nanoparticles, has shown promising results for cancer immunotherapy in animal models (60). Due to the slow release of the antigens from these delivery systems, a continuous activation of CD4^+^ and CD8^+^ T-cells is obtained, improving survival rates due to the tumor growth inhibition (61, 62). Other studies with these nanocarriers including ovalbumin (OVA) as antigen model have been also performed, showing encouraging results *in vivo* that can be interesting against infectious diseases (63).

A second approach for targeting DCs is the genetic engineering, which allows fusing the selected antigen to a single-chain fragment variable for the target receptor (64). This approach has been widely utilized for the delivering of wide range of pathogen-derived antigens to different receptors of DC both *in vivo* and *in vitro* (65–67). For instance, the use of genetically engineered mAb against DEC-205 receptor has been demonstrated to activate CD4^+^ and CD8^+^ T-cells responses and to induce protective immunity against different infectious agents such as *Leishmania major* (68), *Yersinia pestis* (69) or viral infections like recombinant vaccinia virus or HIV-1 (70). So far, the targeting of DEC-205 receptor is the only one that has progressed into clinical trials for cancer research (71). Nevertheless, other receptors, such as DC-immunoreceptor (DCIR) or CD40 have been also targeted with genetically modified mAb fusion to HIV-1 antigens, demonstrating their safety and antigenicity in mice and non-human primates (NHPs) (72). Targeting HIV-1 Envelop (gp140 ZM96, gp140z) to DC through the CD40 receptor is currently under phase I/II clinical evaluation (NCT04842682). Finally, the conjugation of the antigens to mAb can be assessed by non-covalent binding. For instance, a system based on dockerin-cohesin bacterial proteins, which interact with high affinity and specificity, has been studied for HIV-1 (73) or influenza vaccine (74). The study demonstrated that the system could form a stable antigen-antibody complex, and that the vaccine elicited -specific Ab and T-cell responses in mice when immunized with the HA1 subunit of influenza hemagglutinin conjugated to anti-CD40 mAbs. Recently, these authors have used also the same dockerin-cohesin system with HIV-1 Env (gp140z) antigen, for targeting LCs *in vivo*, demonstrating the increase of antigen-specific B-cell responses in mice after immunization (75). Thus, this system appears to be useful for the development of prototype vaccines when antibody fusion to antigen cannot be expressed. Other non-covalent systems to assembly the mAb to different targeted treatments have been also recently developed. These are based on the linkage between a Fc-binding proteins that carry the drug, such as recombinant staphylococcal Protein A or Protein G, and the mAb (76).

### DC receptors

When targeting antigens to DCs *in vivo*, the specificity of the receptor has to be taken into account, since i) targeting DCs *via* distinct lectins leads to different types of immune responses (77), and ii) the receptor might be shared by multiple cell subsets (78). For instance CD11c, the classical DC-marker in mouse, is expressed on activated CD8^+^ T-cells (79), NK cells (80) and macrophages (81). In addition, the receptor can be shared by different DC populations, which drive specific immune responses due to their functional plasticity (82). In the last decades, different DC receptors have been studied for vaccine targeting, including Fc receptors (FcR), CD11c, LOX1, CD40, DCIR or C-type lectin receptors such as DEC-205 (CD205), DC-SIGN (CD209), mannose receptor (CD206), Langerin (CD207) or DNGR1/Clec9A or XCR1, among others (83). From these, CD40 and DEC-205 have been the most widely used for cancer and infectious diseases and moved into clinical development (84). Even though it is also present on monocytes and at low levels, on T and NK cells (85), DEC-205 has shown very promising results for HIV-1 vaccination studies. Thus, the use of an anti-DEC-205 mAb fused to HIV-1 Gag p24 induced strong T-cell response in mice (83) and NHP when combined with poly(I:C) or Poly-ICLC (Hiltonol^®^) (86). Conversely, other studies have demonstrated that the targeting of DC through DEC-205 receptor without any adjuvant leads to the induction of tolerance (87). LOX-1 or Lectin-like oxidized low-density lipoprotein (LDL) receptor-1 also appeared to be a promising target for HIV vaccination. An anti-LOX-1 mAb fused with Env gp140z fusion protein elicited robust cellular and humoral responses in primates when co-administered with Poly-ICLC (Hiltonol^®^) (88). Furthermore, an anti-LOX-1 specific antibody fused to influenza virus haemagglutinin 1 (HA1) injected to macaques showed higher levels of HA1-specific neutralizing antibodies and had reduced viral titers following subsequent infection with influenza virus when compared with animals immunized with the inactivated influenza virus (35).

Langerin or CD207 receptor has been also studied for dermal vaccination. This surface molecule is mainly expressed in a certain subset of LCs and dermal DCs and its *in vivo* targeting with foreign antigens fused to mAb induced CD8^+^ T-cell proliferation (89). Specifically, mAb conjugated with OVA or HIV-1 peptides have shown promising results after vaccination (90). For instance, HIV-Gag antigen fused to an anti-Langerin mAb intradermally injected to NHP induces LC activation and migration out of the epidermis, and improves anti-HIV-1 immune response without adjuvant (91). In addition, other studies using *Staphylococcus aureus* infection-mouse model have demonstrated the LC-induced humoral responses, obtaining specific IgG1 levels after patch immunization (92). Finally, a novel LC-targeting DNA vaccine platform has increased the list of strategies for DC-targeting immunotherapies. Upon topical patch-mediated immunization of ‘pathogen-like’ nanoparticles, LCs are capable to uptake the antigens and accumulated them in the nuclear region, showing an effective DNA delivery *in vivo*. In addition, studies on tissue distribution revealed that the DNA was delivered into the lymph nodes, demonstrating the migration of LCs to the immune organs, and appearing as an attractive approach for intradermal vaccine delivery (93).

CD40 is a potent activating receptor expressed by a range of APCs, including DCs, B-cells and macrophages (94). CD40 signaling induces DC maturation and plays an essential role in connecting innate and adaptive immunity. Its presence on the surface of DC promotes cytokine and chemokine production, induces expression of costimulatory molecules, and facilitates the cross-presentation of antigens. Furthermore, it is necessary for T-cell-dependent humoral responses and therefore to promote antibody development (95).

**Figure 2:**
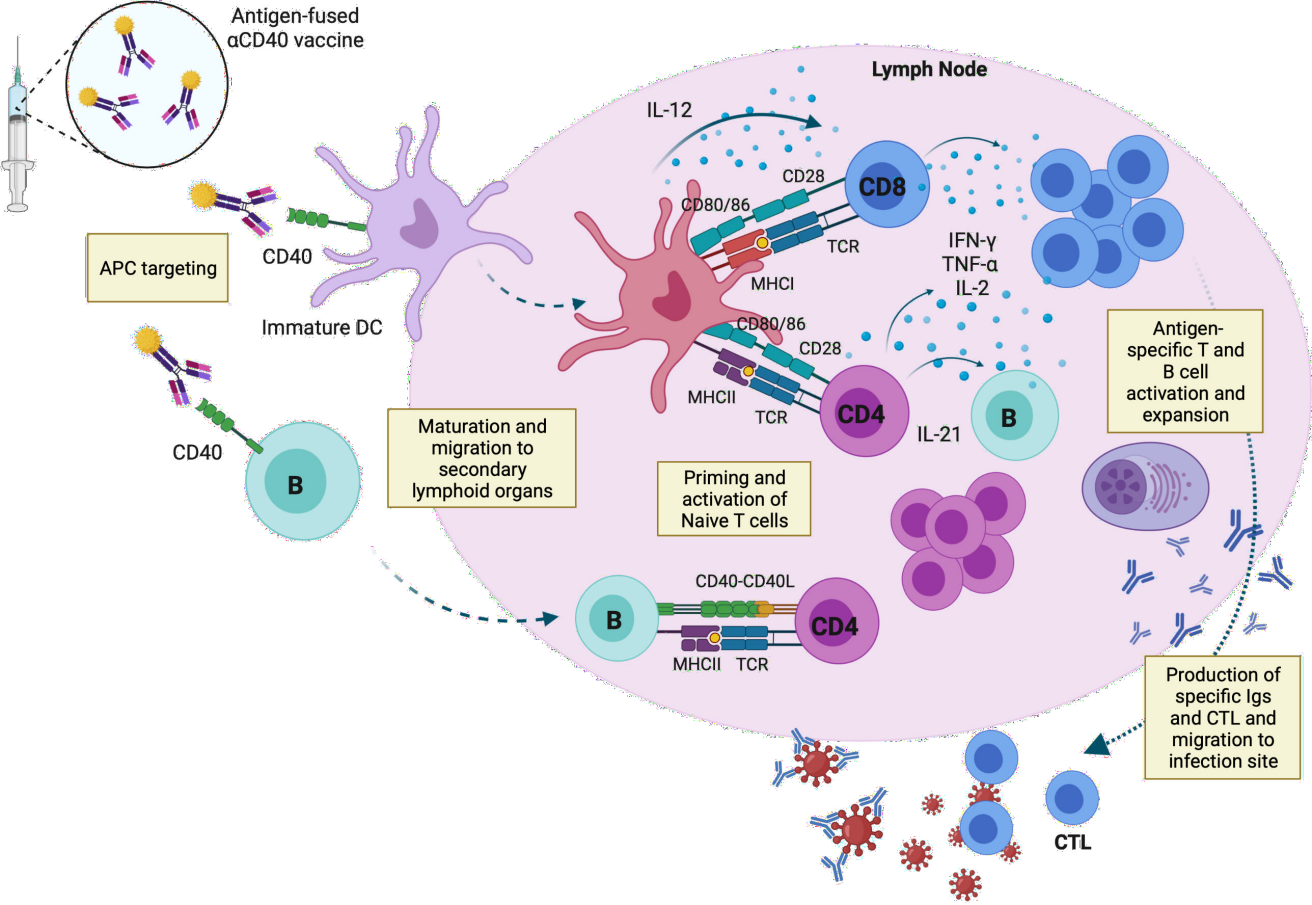
Capture of the anti-CD40 vaccine by APCs and activation of T- and B-cell responses in the draining lymph node. Targeted vaccines recognize CD40 molecules expressed on the surface of immature DCs and B-cells. The vaccine induces the maturation and migration of the immune cells to secondary lymphoid organs, where they present the peptides to naïve T-cells through MHC-I and -II complexes. Mature DC release IL-12, which stimulates the differentiation and expansion of T-cells, which in turn release pro-inflammatory cytokines such as IFN-γ, TNFα or IL-2, promoting the activation of cytotoxic T-cells. B-cells can also present the antigen to naïve CD4^+^ T-cells through CD40-CD40 ligand, inducing maturation and proliferation of antigen specific T-cells, which trigger B-cell maturation by IL-21. Antigen specific antibodies and T-cells migrate to the infection site to neutralize the virus and face the pathogen. APC, antigen-presenting cells; IL, interleukin; CTL, Cytotoxic T lymphocyte; TCR, T-cell receptor. This image was created with BioRender software.

Regarding B-cells, CD40 ligands provides them a survival signal, which leads to B-cell longevity and differentiation to plasma cells. Like DCs, CD40-activated B-cells migrate to secondary lymphoid organs where they can also present the antigen to CD4^+^ T-cells, and together with DCs support the immune response releasing pro inflammatory cytokines such as IFN-γ, IL-6 or TNFα (96) (**Figure 2**).

Several CD40-targeting vaccines have been demonstrated to be immunogenic against different infectious diseases (**Table 1**). For HIV-1, CD40-targeting vaccination administered to HIV-1-infected humanized mice with poly(I:C) induced HIV-1-specific CD8^+^ T-cells, reduced the HIV-1 reservoirs in lymphoid tissues and induced human IgG production (67, 98). For other viral infections such as Respiratory Syncytial Virus (RSV), the fusion of a viral protein with CD40 ligand delivered by an adenoviral vector into BALB/c mice effectively protected animals against the viral infection, inducing neutralizing antibodies and memory CD8^+^ T-cells (103). The same strategy of recombinant adenovirus vaccines encoding CD40 ligand fused to viral antigens to target CD40 has also been showed to be promising for influenza, demonstrating the capacity of this system to induce a robust and long-lasting protective memory immune response against the virus (101). Other proteins have been also fused to anti-CD40 mAb, such as influenza matrix protein-1 (FluM1), eliciting human specific CD8^+^ T-cell responses, and showing strong immunogenic response of CD40-targeted vaccines compared to anti-DEC-205, DCIR, and Dectin-1 mAb fused to the same antigen (100). Similar results were also obtained for Human papillomavirus (HPV) cancer vaccine, where the recombinant fusion protein of the humanized antibody to CD40 fused to HPV16.E6/7 (αCD40-HPV16.E6/7) antigens evoked HPV16.E6/7-specific CD8^+^ and CD4^+^ T-cell responses in head-and-neck cancer patients *in vitro* and in human CD40 transgenic (hCD40Tg) mice (102). A recent work showed that an anti-CD40 antibody fused to the receptor-binding domain (RBD) of the SARS-CoV-2 spike protein induced significant levels of specific T- and B-cells, with a long-term memory phenotype in a humanized mouse model and the recall of neutralizing antibodies in SARS-CoV-2-convalescent non-human primates after one single dose of the vaccine administered without adjuvants (66). Finally, our lab showed that the fusion of CD40 targeting vaccine to a new generation of B- and T-cell epitopes from Spike and Nucleocapsid viral proteins of SARS-CoV-2 elicited high levels of cross-neutralizing antibodies against different variants in mice, as well as functional and specific T-cells responses *in vitro* (104).

**Table 1:** Anti-CD40 targeting vaccines currently in development against infectious diseases.

Pathogen/Disease	Targeting receptor (TLR ligand)	Antigen	Route	Adjuvant	*In vitro/In vivo* model	Reference
**HIV-1**	CD40/TLR-9	HIV-1 Gag, Nef and Pol (HIV 5pep)	I.m and I.p	CpG-B	Humanized mice	(97)
**HIV-1**	CD40/TLR-9	HIV-Env gp140	I.p	CpG	Humanized mice	(67)
**HIV-1**	CD40/DCIR	HIV-1 Gag, Nef, and Pol (HIV5pep)	I.d	Poly-ICLC	NHP	(71)
**HIV-1**	CD40/TLR-3	HIV-1 Gag, Nef, and Pol (HIV5pep)	I.m and I.p	Poly(I:C)	Humanized mice	(98)
**HIV-1**	CD40/LOX-1/TLR-3	HIV-1 Env gp140	I.d	Poly-ICLC	NHP	(88)
**HIV-1**	CD40/TLR-9 or TLR-3	HIV-1 Gag, Nef, and Pol (HIV5pep)	I.m and I.p	CpG/Poly(I:C)	Humanized mice	(85)
**HIV-1**	CD40	HIV-1 Gag, Nef, and Pol (HIV5pep)	-	-	HIV-infected patient PBMC	(99)
**Influenza virus**	CD40/DEC-205/DCIR/Dectin-1	Influenza matrix protein-1 (FluM1)	I.v	–	Humanized mice	(100)
**Influenza virus**	CD40	Nucleoprotein (NP) and CD40 ligand	I.d	-	CD40L(-/-) and CD40(-/-) mice	(101)
**Influenza virus**	CD40/Langerin	Influenza matrix protein-1 (FluM1)	I.p	–	Human Langerin transgenic mice (huLangerin-DTR)	(97)
**Human papillomavirus (HPV)**	CD40/Langerin/TLR-3	HPV16.E6/7	S.c/I.p	Poly(I:C)	Human CD40 transgenic mice	(102)
**Respiratory syncytial virus (RSV)**	CD40	RSV fusion protein (F) and CD40 ligand	I.n	–	BALB/c mice	(103)
**SARS-CoV-2**	CD40/TLR-3	RBD	I.p/S.c	Poly(I:C)/No adjuvant	Humanized mice/NHP	(66)

I.p., Intraperitoneal; S.c, subcutaneous; I.m, intramuscular; I.n, Intranasal; I.d, intradermal; I.v, intravenous.

Among all the DC-receptors, CD40 appears to elicit superior T-cell responses compared to others. This might be explained due to the target and accumulation of the antigens within the early endosome compartment, which facilitates cross presentation compared to other receptors such as DEC-205, which drives the antigens to late endosomes (105). Other studies also show that this receptor is the most efficient at priming and boosting antigen specific CD8^+^ T-cells *in vitro* and *in vivo* (74), what makes it attractive since the induction of specific CD8^+^ cytotoxic T-cells is still a challenge for the success of some vaccines under development (17).

Different clones of anti-CD40 mAb have been developed up to date. Flamar *et al.* demonstrated in 2013 the efficacy of the humanized anti-CD40 12E12 mAb, a chimeric IgG4 fused to five different HIV-1 peptides produced in mammalian cells. This clone was able to induce specific memory CD4^+^ and functional cytotoxic CD8^+^ T-cells (99). Several CD40-targeting vaccines based on 12E12 are currently under development, including anti-CD40-HPV16 E6/E7 (102), anti-CD40-HIV-1 Env (gp140z) (106), anti-CD40-HIV5pep (72), and anti-CD40 coupled to SARS-CoV-2 proteins (66), demonstrating safety and efficacy on different animal models such as human CD40 transgenic mice, humanized mice or NHPs (65).

Recently, these authors described the kinetic parameters and affinity constants for binding of the different anti-CD40 IgG4 mAb available nowadays, showing that the different antibodies bind with high affinity to CD40 receptor. Interestingly, they showed that some antibodies such as 12E12 or 12B4 prevented CD40 binding to CD40 ligand (CD40L), fully blocking the required sites for this attachment, unlike the 11B6, 24A3, CP or S2C6 antibodies, which had a minimal effect on CD40L (65). However, this fact does not appear to correlate with activation potential (107). The *in vitro* cultures of PBMCs from HIV-1-infected donor, treated with anti-CD40-CD40L-HIV5pep vaccine have validated the importance of anti-CD40 12E12 clone to induce antigen-specific CD4^+^ T-cell responses compared to anti-CD40 11B6 clone which was capable to induce stronger antigen specific CD8^+^ T-cell responses. In these same cultures, anti-CD40 11B6 and 12E12 clones were similarly capable at expanding Flu M1-specific CD4^+^ T-cells (65). In fact, this and other humanized mAb have the advantage of not being immunogenic *per se*, what avoids unspecific immune responses, and to have good stability, allowing their manufacturing and scale-up. In April 2021, the 12E12 anti-CD40 HIV-1 Env gp140z vaccine co-administered with Poly-ICLC adjuvant (Hiltonol^®^) moved into clinical phase I study in healthy volunteers (ANRS/INSERM/VRI 06 study).

## Epitope mapping for DC vaccine development

### *In silico* down-selection of the best-in-class vaccine antigen

In the last years, peptide-based vaccines have appeared as a new antigenic strategy for vaccine development. These vaccines have a fully defined composition and constitute an affordable approach for large-scale production: they are stable upon storage, with no biological contamination, minimum allergenicity or autoimmune responses and can be used as therapeutic or prophylactic means. A benefit of DC-targeting platforms is to specifically address selected epitopes to DC to improve internalization of the antigens, processing, and initiation/stimulation of the immune responses. Therefore, in addition to the full-length antigens, the possibility of exploring the immunogenic epitopes of these proteins to be targeted to DCs may offer some advantages, such as the lower antigen complexity which makes easier to combine multiple different epitopes from different proteins to induce highly specific protective immune responses or the decrease of the risk of unwanted cross-reactions. However, the selection of these epitopes needs an appropriate identification and following evaluation. For that, different *in silico* epitope-mapping strategies must be used to choose an epitope that is well-conserved among pathogen species or strains, presents high affinity binding to HLA molecules, which is stable, immunogenic, able to induce memory responses, covers B and T-cell epitopes and which has non-allergenic properties. Thus, when designing these vaccines, the composition of antigenic molecules and the competitive high-affinity binding to MHC molecules should be considered. A relative balance between immunodominant and immuno-prevalent T-cell epitopes needs to be settled. While immunodominant epitopes elicit the best immune response, they might present high response variability among individuals (108), potentially generating an unequal vaccine performance. In a case of a mutation, it will become ineffective. There are many immunodominance determinant factors, regarding antigen and T-cell related factors. Among the first ones, we find its affinity to MHC molecules, stability, processing and transport, kinetics of transcription and translation. For those depending on T-cells, the time needed for CTL clonal expansion, T-cell precursor frequency, TCR repertory, its affinity and avidity, the strength of the signal, proliferative capacity, intrinsic ability to respond, competition for resources, among others (109). Immuno-prevalent T-cell epitopes are mostly immunogenic in the condition of various alleles. They are more common across individuals with different HLA type and can induce specific-IFN-γ responses by high T-cell responding frequency within the repertoire.

Conventional approaches for the identification on immunogenic epitopes are time consuming and extremely laborious and *in silico* predictions can decrease the number of experiments needed (110). Thus, immunoinformatic tools take into consideration the host immune reactions, providing further approaches in vaccine design against different diseases. These tools are cost-effective, convenient and help as preliminary study prior to the *in vivo* validation studies (111). So many databases and algorithms are available nowadays for the screening of B and T-cell epitopes (cytotoxic T lymphocyte, CTL; and helper T lymphocyte, HTL). These tools allow protein sequences screening and the identification of MHC binding aggregates and the best motifs to be used among human populations with genetic variability.

Several databases exist providing a wide range of information for the identification of protein sequences antigenicity, their structural modulation, IFN-γ inducing epitopes, allergenicity, physicochemical properties, stability, molecular docking, codon adaptation and *in silico* cloning.

### T-cell epitope prediction and immunoinformatics: direct and indirect methods

T-cells scan MHC-bound ligands. This allows them to detect the antigens originated from microorganisms as well as the presence of aberrant self-antigens. These complexes (MHC/ligand) result in a chain of enzymatic events involving distinct specialized organelles and pathways depending on whether the signal comes from the MHC-I or –II molecules. While the molecules coming from the interior of each cell are sampled on class I molecules, MHC-II mostly presents peptides from the extracellular environment. The MHC-bound ligands that provoke a T-cell immune response are called T-cell epitopes.

Different methods can be used to predict T-cell epitopes. The direct way, based on the prediction of T-cell receptor (TCR) recognition, with sequential and structural analysis the epitopes; and the indirect one, which relies on the prediction of MHC/HLA binders developed generally into the two different groups MHC class I and II binders, which is more accurate and specific compared to the direct method (112).

The goal of MHC binding and MHC ligand processing and elution predictions is to identify T-cell epitopes. They translate the differences in predicted MHC binding affinity related to T-cell recognition. The comparison between them show that an affinity measurement of IC50 < 500 nM is a valuable threshold to determine ~90% of class I restricted T-cell epitopes (113).

Analysis of the Immune Epitope Dataset (IEDB) confirmed the usefulness of 500 nM as a general threshold that captured about 85% of all the epitopes when epitopes from all alleles were considered together (114). For MHC-II molecules, an IC50 < 1,000 nM threshold is settled based on this same methodology as for class I threshold (115).

The endogenous antigen processing goes through different steps: its recognition, proteasome cleavage into smaller fragments, their transportation through the transporter-associated with antigen processing (TAP) protein complex to the endoplasmic reticulum and finally their presentation by MHC-I. In this respect, PSCs (116) and Netchop 3.1 (117) programs were developed in order to predict the T proteasomal cleavage sites, in addition to TAPPred (118), and TAPhunter (119) that were established for the prediction of the binding affinity of these antigens towards TAP complexes.

Several advanced immunoinformatic tools for T-cell epitopes prediction exist, including MHCPEP, SYFPEITHI, AntiJen, MHCBN, EPIMHC, IEDB, IMGT/HLA, MHCPred, Epivax, RANKPEP, EpiJen, nHLAPred, ProPredI, MMBPred, NetMHCpan, NetCTL, IEDB MHC-I binding, BIMAS, MHC2Pred, IMTECH, Propred, NetMHCII, NetMHCIIpan, IEDB MHC-II binding and NetMHC with superior performance (120). Some others are more pathogen- and tumor- specific databases such as DFRMLI, CIG-DB, CTDatabase (121), AntigenDB, Protegen, HIV Molecular immunology database, HCV immunology database and TANTIGEN (122). IEDB is the largest and most complete epitope database (123), comprising both epitope and assay information concerning epitopes from different infectious, allergic and autoimmune diseases, in addition to alloantigens for primates, humans, mice and host species (113).

Considering MHC-I binders T-cell epitopes prediction methods, many tools have been developed to date. In addition to MixMHCpred20.1 that showed very good performance in the extensive benchmarking tests, one consensus-based server achieving the most accurate predictions is the NetMHCcons1.1, which integrates three algorithms NetMHC, NetMHCpan and PickPocket. While the first two are based on Artificial Neural Network (ANN) method, Pickpocket is a matrix-based way relying on receptor-pocket similarities among MHC molecules. NetCTL pan 1.1 is another quantitative matrix-based method of prediction in protein sequences assembling peptide MHC-I binding prediction, TAP transport efficacy and proteasome C-terminal cleavage. Another accurate system is the nHLAPred based on both quantitative matrix and ANN method.

The prediction of MHC-II binders is more complicated since their groove structure is open, in contrast to MHC-I. Among direct methods for the prediction of HTL epitopes, IFN-epitope has been recently used to forecast and design IFN-γ inducing peptides (124), MHC-II binders and T-cell epitopes, in addition to PREDIVAC, which showed significant improvements comparing to prior methods (125).

Indirect methods predicting MHC-II binders are mostly based on machine learning techniques such as ANNs, support vector machines (SVMs) and hidden Markov models (HMM). Furthermore, ProPred (126), EpiDock (127) and NetMHCIIpan3.1 (128), can also predict the binding of MHC-II molecules for both human and mice. Two main issues affect HLA-binding predictions accuracy: first, the MHC class-I and-II alleles for which predictions are available; and second, how sophisticated and sensitive these predictions are (129). Thus, to clearly guide users for what methods and threshold apply for their predictions, the performance of these algorithms should be revised for their capacity of T-cell epitopes identification in large-scale data sets, screen various alleles and to be generated consistently (120). Researchers must select suitable prediction tools that best adapt their objectives. Different studies used this reverse vaccinology strategy in order to select the best in-class epitopes for different infectious diseases such as SARS-CoV-2 (104), Nipah virus (130), *Leishmania* (131), HBV (132), HIV (133), Influenza (134), Tuberculosis (135), Ebola virus (136), *Neisseria* (137), *Plasmodium* (138), *Trypanosoma* (139), or Chikungunya and Mayaro viruses (140).

### Antigen processing predictive approaches

Processing the antigen is a very critical step that indicates T-cell epitopes immunogenicity (141). Available computational tools, which model antigen processing, improve T-cell epitope predictions comparing to only predicting peptide-binding to MHC molecules (142). Taking into account the differences between MHC class-I and II molecules antigen presentations, class-II endocytic antigen derived molecules miss good predictions algorithms since it is not very well-known yet (143).

After their degradation in the cytosol, MHC-I antigens are transported by TAP to the reticulum endoplasmic where they undergo trimming before being loaded onto emerging MHC-I molecules. Many computational tools predicting both proteasome cleavage and TAP peptide binding have been described. For the former, prediction models were derived from peptide slices generated *in vitro* by human constitutive proteasomes, in addition to sets of MHC I-restricted ligands plotted onto their root protein. For the latter, they are based on algorithms of peptides with recognized affinity to TAP. Hence, multiple steps tools combining all these different prediction methods of CTL epitopes were designed such as Propred-1 (144), Epijen (145), PEVAC (146) or MAPPP (147).

### B-cell epitope prediction and immunoinformatics: Linear and conformational approaches

Antigenic determinants, known as B-cell epitopes, are the antigen portion that bind to the immunoglobulin or the antibody. These epitopes might be any exposed solvent region in the antigen and even if mostly proteins, they have different chemical nature. Their recognition is of key importance for the activation of memory B-cells. They have a very challenging role in the development of vaccines against exogenous microorganisms. The main issue is their nature, which could be linear or conformational, in addition to their high variable epitope length. Therefore, prediction of B-cell epitopes is more complex than that for T-cell epitopes (148).

In fact, immunoglobulins that recognize linear epitopes can recognize denatured antigens, while the denaturation of the antigen leads to the loss of recognition for conformational B-cell epitopes. Note that, the majority of B-cell epitopes are conformational and, only a minority (~10%) of original antigens comprises linear B-cell epitopes (149).

However, the prediction of these linear epitopes has obtained extra attention. Different tools and methods exist for their prediction, with those based on machine learning techniques, *e.g.* ABCpred (150) and BepiPred (151), surpassing those focusing on amino acid scales (152) such as PEOPLE (153) and PREDITOP (154). Recently, Wang Tao and coll (155). developed BepiTBR, a linear B-cell epitope prediction tool that is based on the T-B reciprocity, showing the highest chance of a specific B-cell epitope to be nearby a CD4^+^ T-cell epitope.

The prediction of conformational B-cell epitopes is not as developed as linear ones. This is due to the necessity to know the 3D antigen structure, not commonly available. In addition, the method for selecting these epitopes for a specific antibody is complex since it is based on the use of suitable scaffolds for epitope grafting. However, several tools are currently available such as DiscoTope (156), SEPPA 3.0 (157) and CBTOPE (158), which takes into account both sequence-derived and the physicochemical properties of the epitopes.

### Variability of antigens and the down-selection of conserved regions

**Figure 3:**
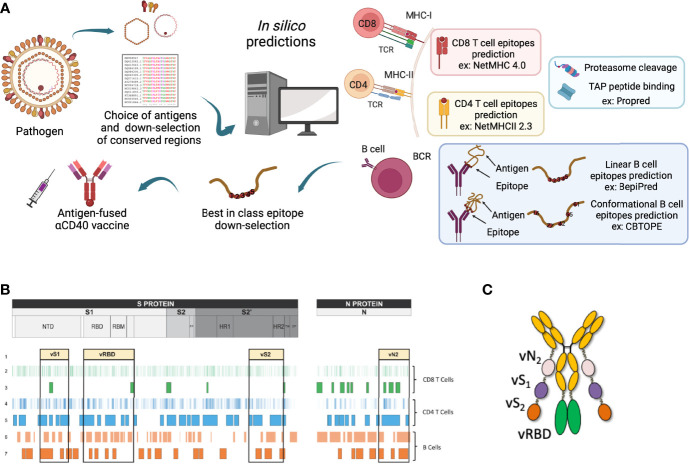
Epitope mapping for the selection of the best-in-class DC-targeting vaccine. **(A)** Viral antigens are chosen depending on current bibliography and what was described at the time of vaccine region selection. This choice takes into consideration the conservation between the different existing viral strains. Selected antigens then undergo *in silico* predictions using corresponding tools. This figure represents MHC-I and -II for CD8^+^ and CD4^+^ T-cell epitopes respectively, proteasome cleavage and TAP peptide binding, linear and conformational B-cell predictions examples. This image was created by BioRender software. **(B)** Example of the design of the Pan sarbecovirus anti-CD40 targeted vaccine after epitope mapping strategy. **(C)** CD40.CoV-2 vaccine construct, adapted from (104).

Due to the continuous mutations of the viral antigens, as occurs for SARS-Cov2 or influenza, current vaccines might respond less effectively towards the infections. To defend against the different variants, a modification of the vaccine composition or a brand-new vaccine should be advanced. The large number of strains makes it impossible to assess each viral epitope in every variant experimentally, thus highlighting the importance of bioinformatics tools to predict the effectiveness of the vaccines and enlighten the immune clearance system. Time being the crucial factor, epitope-based peptide vaccines or recombinant vaccines might be a very helpful solution. Different studies have been accomplished for the prediction of B and T-cell epitopes, including a recent work from our group, in the context of the development of a Pan sarbecovirus anti-CD40 targeted vaccine candidate against multiple variants of concern of the SARS-CoV-2 (104) (**Figure 3**).

To conclude, fusing the down-selected immunogenic peptides to the DC-targeting vehicle is a decisive step. Indeed, the association order of the peptides but also the nature and the length of the linkers between them impact the antigen processing and presentation. By instance, the amino-acid surrounding the antigenic peptides impacts the proteolysis by the proteasome (159), the TAP transport (160) and the ERAAP aminopeptidase trimming (161). Therefore, the development of *in silico* antigen processing programs is an important tool to control the overall antigenicity of the vaccine construct. During the last decade, our group identified linker peptides with low immunogenicity and demonstrated that they were not interfering with the C-I or C-II antigen presentation of the viral peptides included within the DC-vaccine (99, 104).

## Final remarks

In recent years, the place of innovative vaccines based on the most recent knowledge of the induction/regulation and modulation of the immune response with the aim to elicit an integrated T- and B-cell immune responses against complex antigens has emerged beyond “classical” vaccine vectors (recombinant viruses, naked DNA or long peptides). Targeting antigens to endogenous DC appears as a promising strategy to reprogram the immune system.

First, the DC-targeting platform has been significantly improved. Advances of the last decade in the knowledge of DC biology paves the way for the development of innovative DC vaccines. Monoclonal Abs against a dozen of DC-receptors have been generated today and tested for their contribution to shaping the quality and quantity of immune responses. While targeting a chosen DC-receptor, i) specific DC subsets are mobilized, ii) antigen are processed within specific pathway (74), and iii) the receptor targeted triggers signaling within DC for its maturation and cytokine production. Altogether, therapeutic strategies are founded to promote T- and B-cell immune responses capable to prevent or cure the embattled pathology (77).

Beyond a better knowledge on DC phenotypic and functional properties, the design of mAb has also been improved, with the selection of mAb with intrinsic adjuvant properties. We and others generated and tested *in vitro* and *in vivo* a large panel of anti-CD40 clones (either alone or fused with various antigens). These clones bind with high affinity to the CD40 receptors and can be categorized according to their superagonist properties or the need of a CD40L ligand (or combined adjuvant) to activate the targeted DC (65). Therefore, a panel of clones is now well-characterized in preclinical models for not inducing any inflammation or bystander toxicity, making possible their development in clinics. Recent technologies permit to fully humanize DC-targeting mAbs and test them in humans. The cross-reactivity of most of these clones in preclinical models, such as rodents (transgenic mice), non-human primates (macaques or African green monkeys), and humanized mice is well-documented today (66, 67). The identification of cross-reactive DC-targeting mAb to the DC receptors expressed in farm animals potentially open promising prospects for any veterinarian DC-based vaccine approaches (162).

Whilst potent mAbs serve as vehicle to bring the vaccine antigen to the APC (DC but also B-cells), new generations of DC-targeting vaccines are developed today, as recently illustrated by the pan-sarbecovirus DC-targeting vaccine (104). First, a myriad of bioinformatics tools with predictive programs accurate for the selection of the most immunogenic antigens are accessible. One major criteria for down-selecting these immunogen from the full-length protein aa sequence is a high density of class-I and -II T-cell epitopes covering more than 99% of the HLA-I or -II molecules of the worldwide population. As aforementioned, *in-silico* selection is refined by using additional biochemical and immunological predictive programs. Therefore, this technology allows to extend/diversify the number of antigens contained in DC-targeting vaccines, optimizing the antigen presentation, and limiting the amount of antigen, which is of great importance for clinical development, since it may significantly limit the production cost.

The vaccine development requires pathway from discovery through clinical development involving the industry and the public sector and as such the scientific community will benefit from the knowledge and expertise gained with DC-targeting vaccine platforms. DC-targeting technology offers solutions by potentially increasing efficacy while decreasing manufacture costs and complexity, *via* GMP manufacture of a single vaccine that is expected to be administered in low to sub-milligram amounts. The know-how developed in GMP batch production (for HIV-1 and HPV by instance) paves the way for other vaccines targeting other pathogens. To conclude, recent advances in the DC-targeting platform place it as a universal technological platform, with a critical aptitude to be highly reactive and leader in the vaccine development against present and future emerging diseases.

## Author contributions

YP, NG, AH, MC, SC and YL wrote and have a critical review on the Ms. All authors contributed to the article and approved the submitted version.

## Funding

This work has received funding from (i) INSERM and the Investissements d’Avenir program, Vaccine Research Institute (VRI), managed by the ANR under reference ANR-10-LABX-77; (ii) the ANR EBOVAC program under reference ANR-15-CE18-0030, (iii) the French Ministry of Higher Education, Research and Innovation for the Nipah virus project and the SARS-CoV-2 vaccine project; (iv) the European Union’s Horizon 2020 research and innovation programme under grant agreement N° 847939 (IP-cure-B project).

## Acknowledgments

We thank Drs Sandy and Gerard Zurawski for their contribution to the DC-targeting programs and Andres M. Salazar (Oncovir, Inc.) for providing Poly-ICLC (Hiltonol^®^).

## Conflict of interest

The authors, MC, SC and YL, are named inventors on patent applications based on this work held by Inserm Transfert. Inserm Transfert provided a license of CD40 targeting vaccinesto biotech company LinkinVax.

The remaining authors declare that the research was conducted in the absence of any commercial or financial relationships that could be construed as a potential conflict of interest.​​​​​​​​​​​​​​​​​​​​

## Publisher’s note

All claims expressed in this article are solely those of the authors and do not necessarily represent those of their affiliated organizations, or those of the publisher, the editors and the reviewers. Any product that may be evaluated in this article, or claim that may be made by its manufacturer, is not guaranteed or endorsed by the publisher.
